# Supplementary material for: Observation of naturally canalized phonon polaritons in LiV2O5 thin layers
Source: Nat Commun. 2024 Mar 27;15:2696. doi: 10.1038/s41467-024-46935-z (PMC10973474; doi:10.1038/s41467-024-46935-z)
Supplement: Supplementary file 1 — Supplementary Information [file 41467_2024_46935_MOESM1_ESM.pdf]

Supplementary Information: Observation of Naturally Canalized Phonon Polaritons in LiV_2O_5 Thin Layers

Ana I. F. Tresguerres-Mata[1,4†], Christian Lanza[1,4†], Javier Taboada-Gutiérrez[2], Joseph. R. Matson[3], Gonzalo Álvarez-Pérez[1,4,10], Masahiko Isobe[5], Aitana Tarazaga Martín-Luengo[1,4], Jiahua Duan[1,4,11,12], Stefan Partel[6], María Vélez[1,4], Javier Martín-Sánchez[1,4], Alexey Y. Nikitin[7,8], Joshua D. Caldwell[3,9], Pablo Alonso-González[1,4]*

[1]Department of Physics, University of Oviedo, Oviedo 33006, Spain.
[2]Department of Quantum Matter Physics, Université de Genève, 24 Quai Ernest Ansermet, CH-1211, Geneva, Switzerland.
[3]Interdisciplinary Materials Science Program, Vanderbilt University, Nashville, 37212, TN, USA.
[4]Center of Research on Nanomaterials and Nanotechnology, CINN (CSIC-Universidad de Oviedo), El Entrego 33940, Spain.
[5]Max-Planck Institute for Solid State Research, Stuttgart D-70569, Germany.
[6]Vorarlberg University of Applied Sciences, Research Center of Microtechnology, Austria.
[7]Donostia International Physics Center (DIPC), Donostia/San Sebastián 20018, Spain.
[8]IKERBASQUE, Basque Foundation for Science, Bilbao 48013, Spain.
[9]Department of Mechanical Engineering, Vanderbilt University, Nashville 37235, TN, USA.
[10]Present address: Center for Biomolecular Nanotechnologies, Istituto Italiano di Tecnologia, via Barsanti 14, Arnesano, 73010, Italy.
[11]Present address: Center for Quantum Physics, Key Laboratory of Advanced Optoelectronic Quantum Architecture and Measurement (MOE), School of Physics, Beijing, China.
[12]Present address: Beijing Key Laboratory of Nanophotonics and Ultrafine Optoelectronic System, Beijing, Institute of Technology, Beijing, China.

*pabloalonso@uniovi.es
† *These authors contributed equally to this work.*

Supplementary sections:

Supplementary Note I. Far-field optical characterization of LiV_2O_5

Supplementary Note II. Dielectric function of LiV_2O_5

Supplementary Note III. Analysis of the canalization of PhPs in LiV_2O_5

Supplementary Note IV. s-SNOM imaging of PhPs in LiV_2O_5 thin layers

Supplementary Note V. Group velocity and lifetime of PhPs in LiV_2O_5

Supplementary Note VI. Influence of the substrate in the propagation of canalized PhPs in LiV_2O_5

Supplementary Note VII. s-SNOM images of LiV_2O_5 PhPs launched by circular nanoantennas

Supplementary Note I. Far-field optical characterization of LiV_2O_5

The infrared optical response of LiV_2O_5 single crystals was probed using Fourier Transform Infrared (FTIR) spectroscopy. To evaluate the anisotropic dielectric response, we leveraged far-infrared polarizers aligned to the in-plane a and b crystal axes, which exhibit starkly contrasting optical responses (Supplementary Fig. 1). From these measurements, we were able to precisely model the dielectric function along each axis (discussed in Note II) to determine the optical phonons present, which give rise to the hyperbolic, elliptic, and canalized phonon polaritons observed.

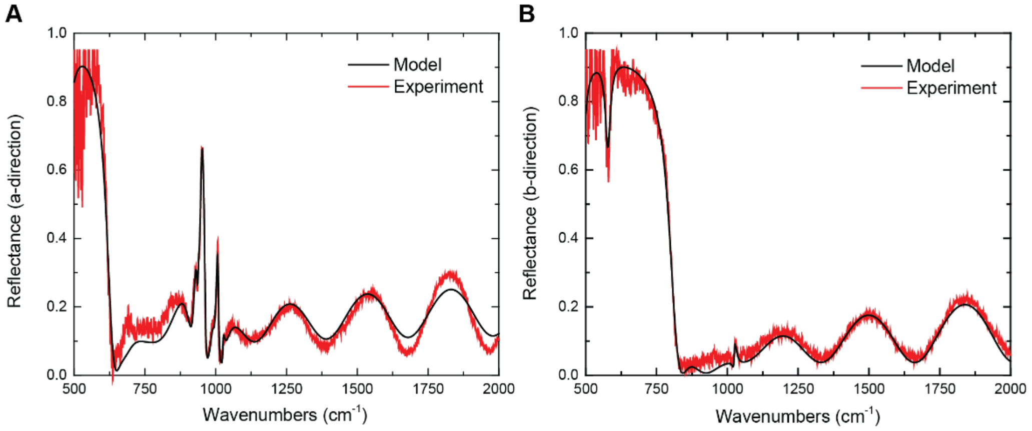

Supplementary Fig. 1. FTIR reflectance spectra of a LiV_2O_5 crystal on a BaF_2 substrate, from $\omega \sim 500\ \mathrm{cm}^{-1}$ to $\omega \sim 2000\ \mathrm{cm}^{-1}$. **A**, **B**, Experimental reflectance spectra (red) and fit (black) along the a and b directions, respectively.

We conducted reflectance measurements (along the bc-axis) at different incident angles for comparative analysis. Typically, a 36x objective is used for FTIR spectroscopy on this type of sample due to its small size. This objective has a nominal incidence of 25 degrees, with an angular spread. The result of this objective is depicted by the black solid line in Supplementary Fig. 2. In addition, results obtained with a 15x Cassegrain objective with

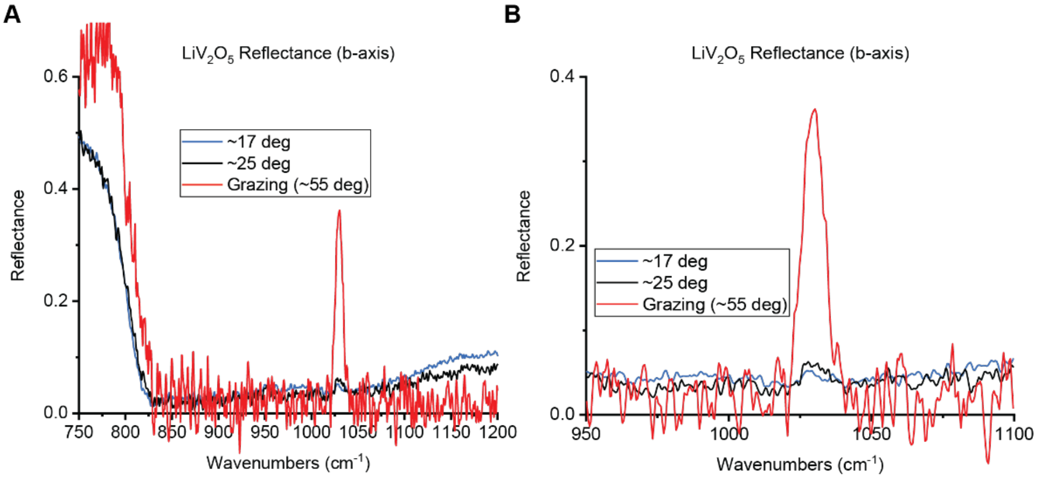

Supplementary Fig. 2. Reflectance FTIR measurements were obtained using three objectives with different nominal incident angles: 36x objective with a 25-degree

incidence (black solid line), 15x Cassegrain objective with a 17-degree incidence (blue solid line), and grazing-incidence objective with a 55-degree incidence (red solid line). **A**, Reflectance measured in the spectral range from 750 to 1200 cm^{-1}. **B**, Zoom-in of **A** from 950 to 1100 cm^{-1}.

a nominal incidence of 17 degrees (blue solid line) and a grazing-incidence objective with a nominal incidence of 55 degrees (red solid line) are also shown.

Supplementary Fig. 2 clearly reveals that the reflectance peak at around 1020 cm^{-1} weakens with decreasing incidence angle, allowing us to corroborate its origin from the response of a c-polarized oscillator. Thus, despite the challenges posed by the thin nature of the sample, which makes it difficult to accurately determine the properties of the out-of-plane phonons, this observation allows us to identify the presence of the c-oriented optical phonon. The provided phonon parameters (reference [34] from the main text) yield a good fit for the dielectric function. We have refrained from including the dielectric function model spectra here, as the other two spectra were unable to fully capture the flake size (due to lower magnification objectives), making it challenging to completely remove the background.

Supplementary Note II. Dielectric function of LiV_2O_5

We have used a Drude-Lorentz (TO-LO) model to compute the dielectric permittivity tensor of LiV_2O_5 according to the measurements described in S1[1]. The TO-LO model for an arbitrary number of oscillators can be written as follows:

$$\varepsilon_i = \varepsilon_\infty^i \prod_{j=1}^{N_i} \left(\frac{\left(\omega_{LO,j}^i\right)^2 - \omega^2 - i\gamma_{LO,j}^i}{\left(\omega_{TO,j}^i\right)^2 - \omega^2 - i\gamma_{TO,j}^i} \right) \quad \text{(S1)}$$

The obtained high frequency permittivities are $\varepsilon_\infty^a = 6.28$, $\varepsilon_\infty^b = 4.48$, and $\varepsilon_\infty^c = 2.71$. In Table S1 we show the obtained values of the TO and LO phonon frequencies and their corresponding damping rates. The first column refers to the principal crystal axis (a, b, or c). The second column denotes the index of the j-th oscillator along the i-th direction.

**Crystal Axis (i)**	**Oscillator Index (j)**	ω_{TO} (cm^{-1})	γ_{TO} (cm^{-1})	ω_{LO} (cm^{-1})	γ_{LO} (cm^{-1})
**a**	1	483.1	27.9	625.2	1.8
**a**	2	731	149	738.9	168.6
**a**	3	948.4	5.5	963.9	9.1
**a**	4	1004.9	6	1011.5	8.5
**b**	1	513.3	21.6	573.3	13.9
**b**	2	581	26.3	795.4	18.6
**c**	1	980	4.8	1024	4.8

Supplementary Table 1. Parameters of the IR dielectric function of LiV_2O_5 obtained by FTIR on a layer with thickness $d = 6.4$ µm. The first and second columns stand for the crystal axis and oscillator index, respectively. The remaining columns show the obtained parameters of the TO phonon frequency ω_{TO}, damping rate γ_{TO}, LO phonon frequency ω_{LO} and damping rate γ_{LO}.

In addition to the study shown in the main text of the permittivity function in the mid-IR spectral range, we have also extracted its dependence on the three crystal directions a, b, and c at lower frequencies ranging from $\omega = 500\ \mathrm{cm}^{-1}$ to $\omega = 800\ \mathrm{cm}^{-1}$ (Supplementary Fig. 3A,B). We observe two spectral regimes of interest: an elliptic regime between $\omega \sim 500\ \mathrm{cm}^{-1}$ and $\omega \sim 625\ \mathrm{cm}^{-1}$ with $\varepsilon_a < 0$ and $\varepsilon_b < 0$, and a hyperbolic regime between $\omega \sim 625\ \mathrm{cm}^{-1}$ and $\omega \sim 789\ \mathrm{cm}^{-1}$ where $\varepsilon_a > 0$ and $\varepsilon_b < 0$.

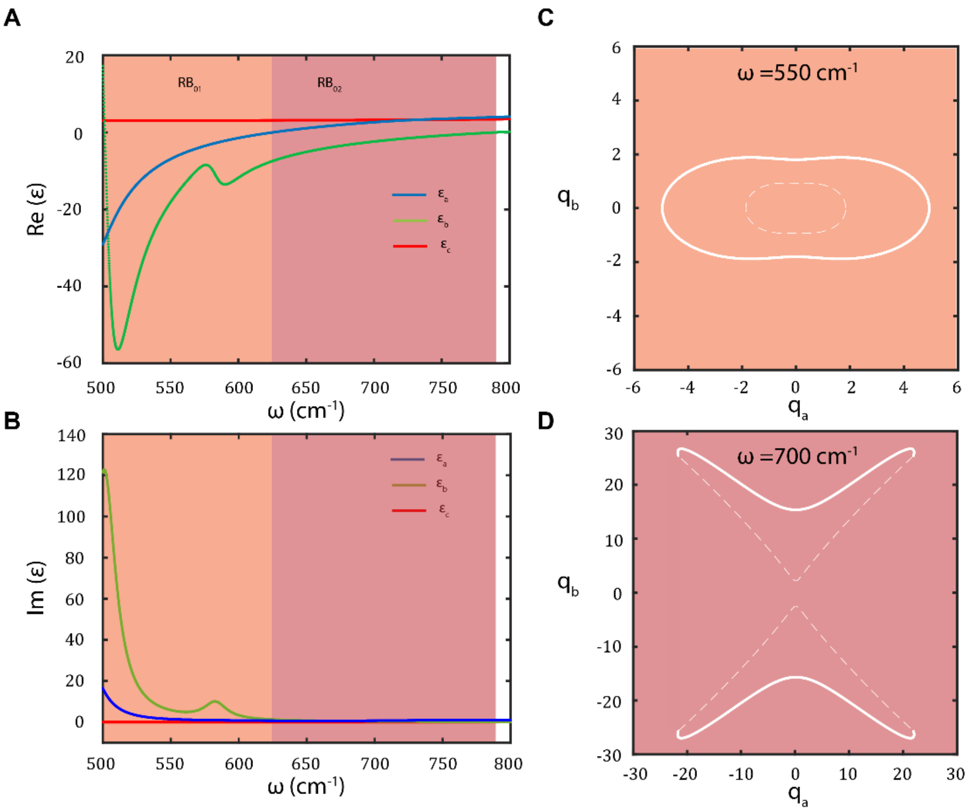

Supplementary Fig. 3. Dielectric function of LiV_2O_5 from $\omega \sim 500\ \mathrm{cm}^{-1}$ to $\omega \sim 800\ \mathrm{cm}^{-1}$. **A**, Real, and **B**, imaginary part of the permittivity function along the a, b, and c crystal directions. Two Reststrahlen Bands are observed: RB_{01} (elliptical, from $\omega < 500\ \mathrm{cm}^{-1}$ to $\omega \sim 625\ \mathrm{cm}^{-1}$) and RB_{02} (hyperbolic, from $\omega \sim 625\ \mathrm{cm}^{-1}$ to $\omega \sim 789\ \mathrm{cm}^{-1}$). In addition, a phononic resonance is observed at $\omega \sim 580\ \mathrm{cm}^{-1}$ along the b direction. **C**,**D**, Analytical IFCs calculated at $\omega \sim 550\ \mathrm{cm}^{-1}$ (RB_{01}) and $\omega \sim 700\ \mathrm{cm}^{-1}$ (RB_{02}). The white continuous lines correspond to the real part of the IFC, while the white dashed lines correspond to the imaginary part of the IFC.

Interestingly, Supplementary Fig. 3C,D show the real (continuous white line) and imaginary parts (white dashed line) of the IFC in RB_{01} (at $\omega \sim 550\ \mathrm{cm}^{-1}$) and RB_{02} (at $\omega \sim 700\ \mathrm{cm}^{-1}$), revealing an elliptic and hyperbolic propagation of PhPs, respectively.

Supplementary Note III. Analysis of the canalization of PhPs in LiV_2O_5

Polariton canalization requires a high flattening of the IFC, which is translated in real space into a highly in-plane collimated or in-plane diffraction-less polariton field. Such a flattening can be studied in LiV_2O_5 by means of the slope of the b component (direction along which the canalization takes place) of the IFC, given by:

$$\frac{dq_b}{d\varphi} = \frac{dq}{d\varphi} sin\varphi + qcos\varphi \qquad \text{(S2)}$$

where, assuming $\varepsilon_s = \varepsilon_s = 1$ for simplicity,

$$\frac{dq}{d\varphi} = \frac{dq}{d\rho}\frac{d\rho}{d\varphi} = \left[\frac{q}{\rho} + \frac{\rho\varepsilon_c}{k_0 d}\frac{2}{\varepsilon_c^2 + \rho^2}\right]\frac{\rho^3}{\varepsilon_c}(\varepsilon_b - \varepsilon_a)sin\varphi cos\varphi \qquad \text{(S3)}$$

The meaning of Eq. S2 is the variation of the normalized polariton wavevector component q_b as the in-plane angle φ changes, i.e., the higher this slope the less the observed flattening (and thus no canalization of PhPs).

Eq. S3 leads to the following version of Eq. S2:

$$\frac{dq_b}{d\varphi} = \left\{q\left[\frac{\rho^2}{\varepsilon_c}(\varepsilon_b - \varepsilon_a)sin^2\varphi - 1\right] + \frac{2\rho^4}{k_0 d}\frac{(\varepsilon_b - \varepsilon_a)}{(\varepsilon_c^2 + \rho^2)}sin^2\varphi\right\}cos\varphi \qquad \text{(S4)}$$

Supplementary Fig. 4 shows the results obtained using Eq. S4 for 3 different frequencies: $\omega = 997\ \mathrm{cm}^{-1}$, $\omega = 1002\ \mathrm{cm}^{-1}$, and $\omega = 1007\ \mathrm{cm}^{-1}$.

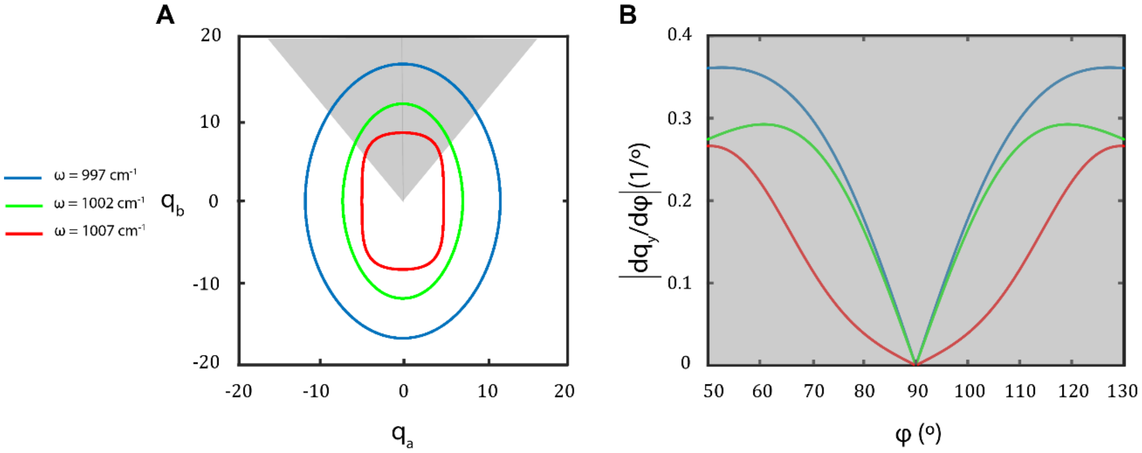

Supplementary Fig. 4. Flattening of the IFCs of PhPs in LiV_2O_5 at RB_{a2}. **A**, IFCs of PhPs in a LiV_2O_5 thin layer at $\omega = 997\ \mathrm{cm}^{-1}$, $\omega = 1002\ \mathrm{cm}^{-1}$, and $\omega = 1007\ \mathrm{cm}^{-1}$. **B**, Calculation of the modulus of Eq. S4 in the gray region of **A**. In all the calculations, the thickness of the flake was chosen to fulfill the experimental data of 213 nm in the main text. Both the superstrate and the substrate have the vacuum permittivity.

Supplementary Fig. 4A shows the IFCs of the PhPs at the 3 different frequencies, observing a clear transition from elliptic to flattened closed ellipse at the canalization frequency $\omega = 1007\ \mathrm{cm}^{-1}$. The gray area has been chosen to compute Eq. S4 in Supplementary Fig. 4B, where the angular domain corresponding to Supplementary Fig. 4A is $\varphi \in [50°, 130°]$. Away from $\varphi = 90°$ (the b direction), the slope of the IFC

dramatically increases in modulus for $\omega = 997\ \text{cm}^{-1}$ and $\omega = 1002\ \text{cm}^{-1}$ in comparison to $\omega = 1007\ \text{cm}^{-1}$.

Supplementary Note IV. s-SNOM imaging of PhPs in LiV_2O_5 thin layers

In this section, we include the s-SNOM measurements performed on two LiV_2O_5 thin layers (thicknesses of 213 nm and 116 nm) from $\omega = 995\ \text{cm}^{-1}$ to $\omega = 1010\ \text{cm}^{-1}$ for the 213 nm flake and from $\omega = 1003\ \text{cm}^{-1}$ to $\omega = 1010\ \text{cm}^{-1}$ for the 116 nm flake. With increasing frequency, we clearly observe a transition from elliptic PhPs to canalized PhPs along the *b* direction (Supplementary Figs. 5 and 6). It is interesting to observe that the Au-antenna for the 116 nm flake is rotated 90º with respect to the other sample, which enables us to confirm canalization, as it occurs along b-direction as well.

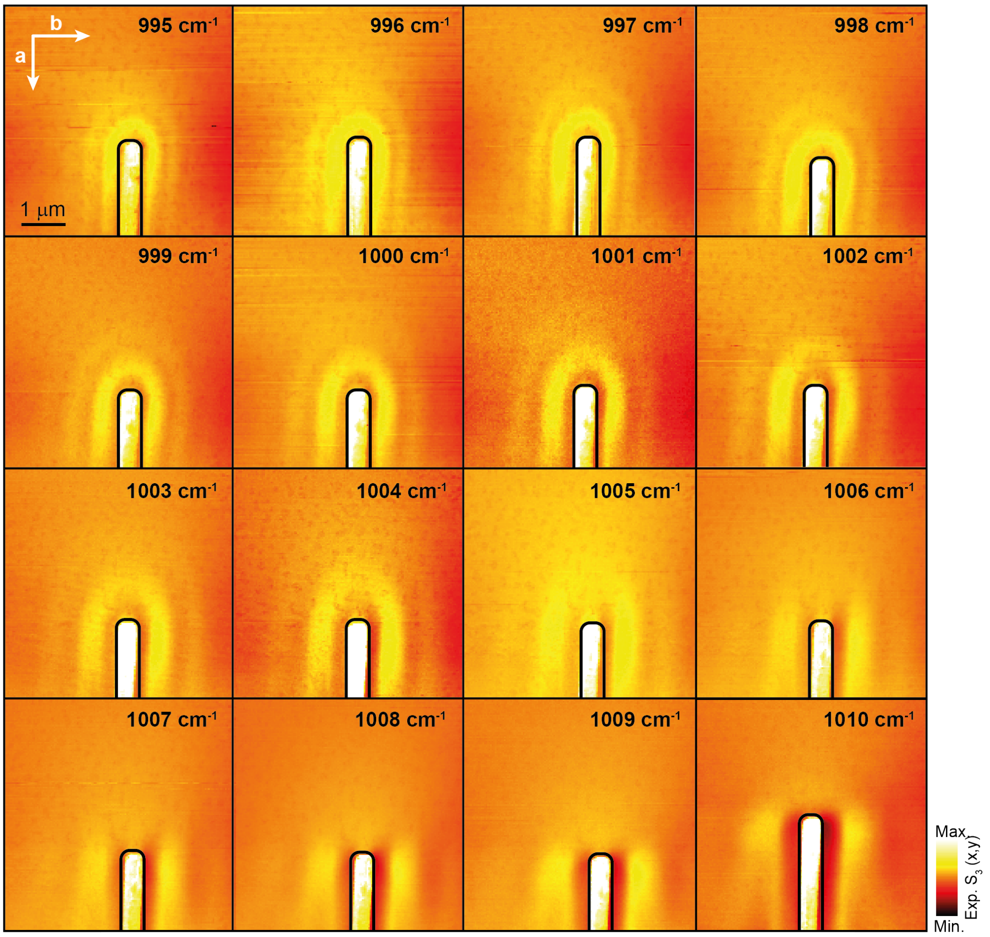

Supplementary Fig. 5. s-SNOM imaging of PhPs in a LiV_2O_5 thin layer (213 nm) from $\omega = 995\ \text{cm}^{-1}$ to $\omega = 1010\ \text{cm}^{-1}$. With increasing frequency, the elliptic propagation of PhPs (launched by a gold antenna) transits to a canalization regime along the *b* direction.

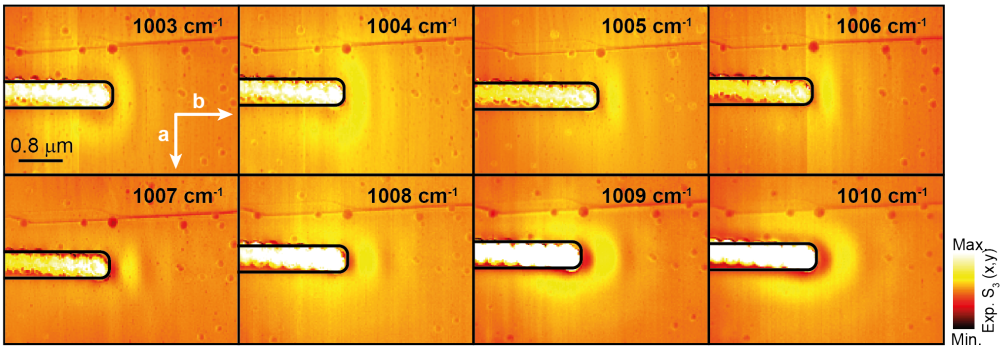

Supplementary Fig. 6. s-SNOM imaging of PhPs in a LiV_2O_5 thin layer (116 nm) from $\omega = 1003\ \mathrm{cm}^{-1}$ to $\omega = 1010\ \mathrm{cm}^{-1}$. With increasing frequency, the elliptic propagation of PhPs (using a 90º rotated Au-antenna) transits to a canalization regime along the b direction.

Supplementary Note V. Group velocity and lifetime of PhPs in LiV_2O_5

The group velocity of PhPs in LiV_2O_5 along the **b** direction has been computed as $v_{gr} = \left|\frac{\partial\omega}{\partial k}\right|$ where ω is the incident frequency and k is the polariton wavenumber[2]. Particularly, we first fit the experimental dispersion (red dots in Supplementary Fig. 7A) to a power function $\omega = a \cdot k^b$ (black curve in Supplementary Fig. 7A) and perform its derivative. The fitting parameters of the power function are $a = 1.15 \cdot 10^3$ and $b = -0.0123$. The derivative (the group velocity) of the fitting in Supplementary Fig. 7A is shown in Supplementary Fig. 7B. A group velocity of about 0.001c (438971.06536 m/s) is obtained at $\omega = 1007\ \mathrm{cm}^{-1}$.

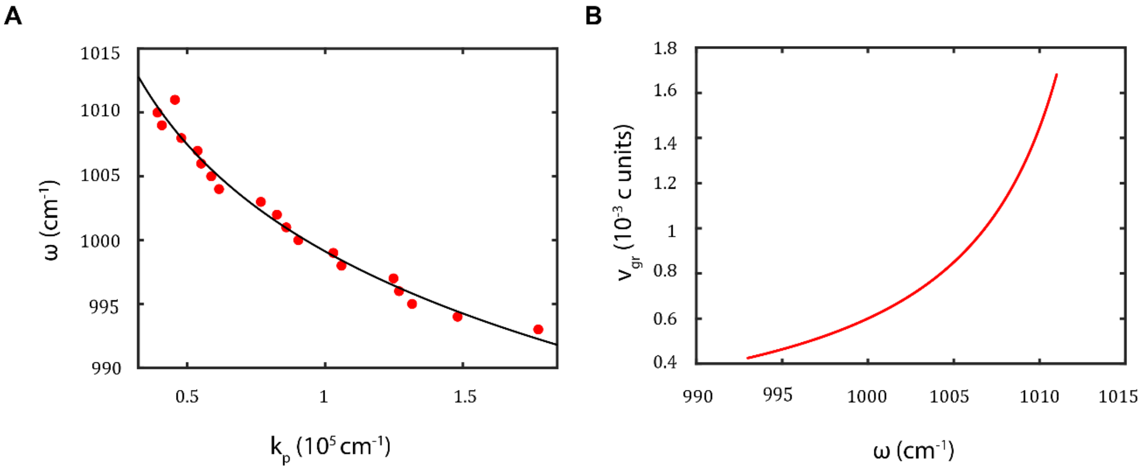

Supplementary Fig. 7. A, Experimental dispersion of LiV_2O_5 PhPs along the b direction. The red dots indicate the experimental data points. The black curve is the fitting to a power function. **B**, Group velocity of LiV_2O_5 PhPs along the b direction. The thickness of the flake fulfills the experimental data of 213 nm in the main text.

The lifetime of LiV_2O_5 PhPs along the b direction at $\omega = 1007\ \mathrm{cm}^{-1}$ was calculated as $\tau = \frac{L_p}{v_{gr}}$, where L_p and v_{gr} are the experimental propagation lengths and group velocities, respectively. The near-field experimental profile (green dots in Supplementary Fig. 8)

was fitted to an exponentially decaying sinusoidal function (red solid line in Supplementary Fig. 8):

$$y = y_0 + Ae^{-\frac{x}{L_p}} sin\left(\pi \frac{x - x_c}{\omega}\right), A > 0, \omega > 0, L_p > 0 \quad \text{(S5)}$$

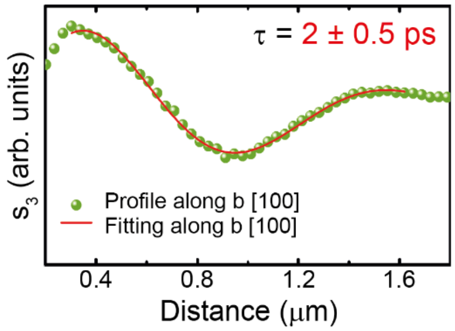

Supplementary Fig. 8. Experimental near-field profile (green dots, s_3) and fitting (red solid line) of LiV_2O_5 PhPs along the *b* crystal direction at $\omega = 1007$ cm^{-1}.

From the fitting of Eq. S5 a propagation length $t_0 = L_p = 0.90$ μm and a wavelength of $\omega = 0.60$ μm were extracted, obtaining a lifetime of $\tau = 2 \pm 0.5$ ps of PhPs along the *b* direction. This lifetime displays the ultra-low-loss nature of PhPs in LiV_2O_5. The same study was performed for the 116 nm flake.

Supplementary Note VI. Influence of the substrate in the propagation of canalized PhPs in LiV_2O_5

Naturally canalized PhPs in LiV_2O_5 have shown to be long-lived (up to 2 ps) and confined about 8 times the free space wavelength for a rather large thickness of the layer (213 nm). These results are strongly influenced by the substrate supporting the LiV_2O_5 layer (SiO_2 through the main text). In Supplementary Figs. 9-11, we compare IFCs, FOM's, group velocities and lifetimes (along the canalization direction) of canalized PhPs in LiV_2O_5 for different substrates[3]. Particularly, we focus on some of the widely used substrates, used both theoretically and experimentally such as SiO_2, BaF_2, SiC, and air. Note that $Re(\varepsilon_{SiO_2}) > Re(\varepsilon_{BaF_2}) > Re(\varepsilon_{SiC}) > Re(\varepsilon_{air})$ at the canalization frequency $\omega =$ 1007 cm^{-1}.

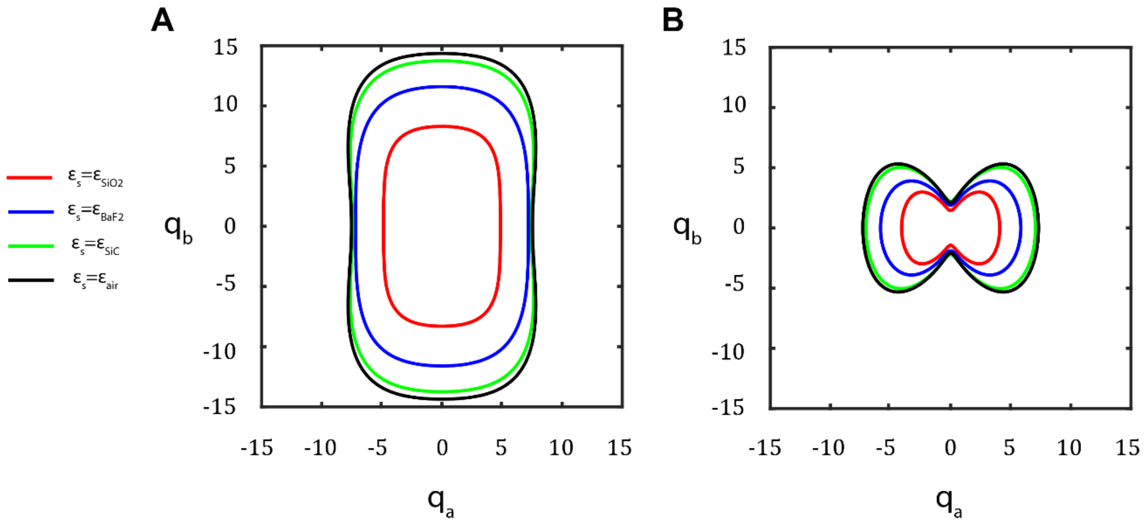

Supplementary Fig. 9. IFCs of PhPs in LiV_2O_5 at $\omega = 1007\ \mathrm{cm}^{-1}$ for different substrates. **A**, Real part of the IFCs. **B**, Imaginary part of the IFCs. In **A**, **B** the IFCs are computed for 4 different substrates: SiO_2 (red), BaF_2 (blue), SiC (green), and air (black). The thickness of the flake is 213 nm.

From Supplementary Fig. 9 we observe that the real component of the IFCs at $\omega = 1007$ cm^{-1} shows a larger confinement (larger wavevector) along both the a and the b directions for the SiC and air substrates. Interestingly, the imaginary component of the IFCs shows a similar value along the b direction for all the substrates, meaning that the relative losses among the different devices are smaller for small permittivity values of the substrate. This feature can be better observed in Supplementary Fig. 10, where the FOM is plotted for both the a and b directions. The PhPs propagate having more in-plane oscillations along the b direction for those substrates with smaller real-part of the permittivity, as can be inferred from Supplementary Fig. 10B. As such, a future suitable platform to enhance the performance of canalized PhPs in LiV_2O_5 could be SiC, due to its close to 1 value of the permittivity and negligible losses in RB_{a2}. Remarkably, the similar values of the imaginary component along the canalization direction imply that the propagation length of the PhPs is similar for all cases, concluding that the substrate does not have a great influence on how long the PhP propagates but only on how many times it oscillates until it (spatially) decays.

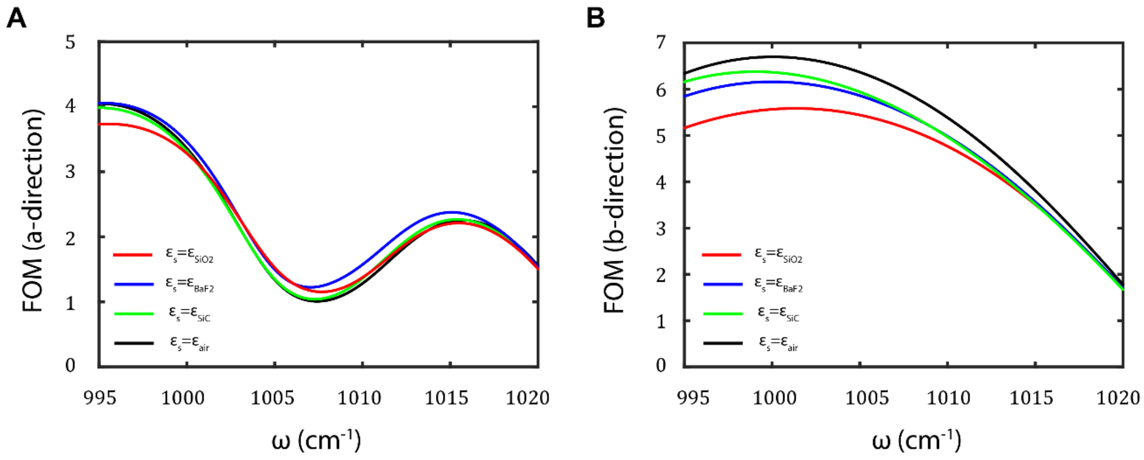

Supplementary Fig. 10. FOMs of PhPs in LiV_2O_5 at $\omega = 1007\ \text{cm}^{-1}$ for different substrates. **A**, FOM along a direction. **B**, FOM along the b direction. In **A**, **B** the FOMs are computed for 4 different substrates: SiO_2 (red), BaF_2 (blue), SiC (green), and air (black). The thickness of the flake is 213 nm. As in the main text, all the FOMs are computed as $\left|\frac{Re(q_p)}{Im(q_p)}\right|$.

In addition to the FOM, we have also computed both the group velocity $v_{gr} = \left|\frac{\partial\omega}{\partial k}\right|$ and lifetime $\tau = \frac{L_p}{v_{gr}}$ for the former devices, as shown in Supplementary Figs. 11A and 11B, respectively. We must note that for the calculation of the lifetime, the propagation length has been calculated as $L_p = \frac{1}{Im(q_p)}$.

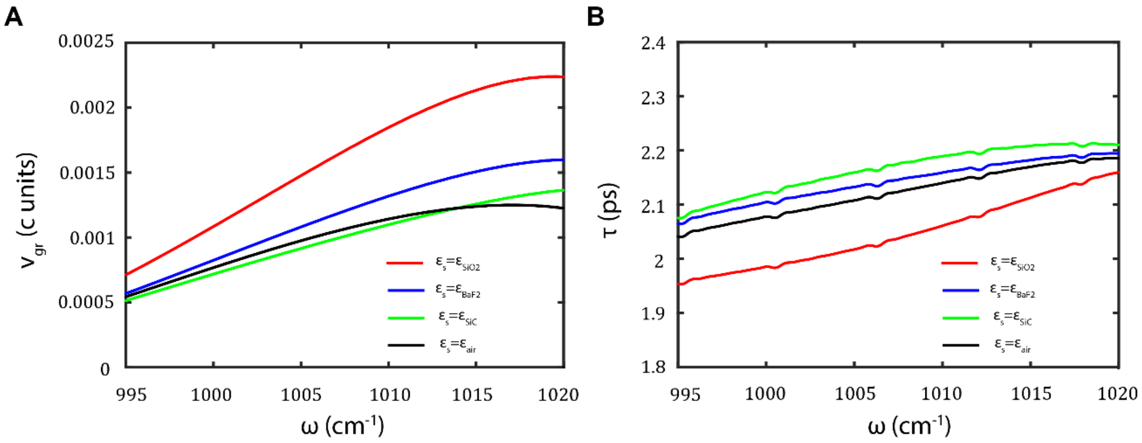

Supplementary Fig. 11. Group velocity (**A**) and lifetime (**B**) of PhPs in LiV_2O_5 for different substrates. All the calculations are computed for 4 different substrates: SiO_2 (red), BaF_2 (blue), SiC (green), and air (black). The thickness of the flake is 213 nm.

As expected, the substrates that lead to better in-plane propagation (SiC and air) also lead to a smaller group velocity as well as a slightly higher lifetime (except for $\varepsilon_s = 1$). The oscillations observed in Supplementary Fig. 11B are due to the numerical differentiation.

In general, using substrates with negligible losses and refractive indexes close to those of free space enhances the polaritonic performance of our devices, not only preserving the unidirectional propagation but also enhancing its lifetime due to the reduction of losses.

Supplementary Note VII. s-SNOM images of LiV_2O_5 PhPs launched by circular nanoantennas

In this section, we show s-SNOM measurements of PhPs in LiV_2O_5 launched by a circular nanoantenna fabricated on top of a single LiV_2O_5 flake. We can observe a clear transition (left to right in Supplementary Fig. 12) in the PhP propagation from elliptical to canalized wavefronts. Although this experiment allows us to unveil natural canalization in LiV_2O_5, the launching efficiency of the circular antennas is clearly not as good as when using a rectangular nanoantenna (Fig. 4 of the main text), where the strong dipolar mode localizes the near fields at its apexes (thus acting as point-dipole sources). In the case of the circular

nanoantennas, the source is no longer point-like but extended, leading to a less efficient launching. Moreover, the surface of the circular antennas is rather rough, which may lead to a decrease in their scattering effectiveness.

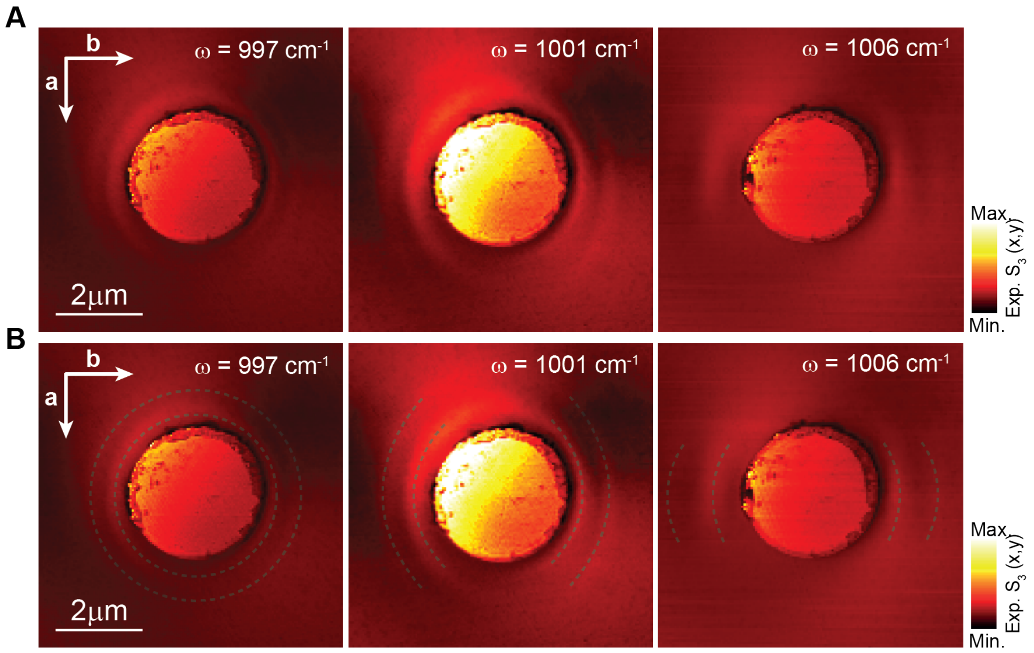

Supplementary Fig. 12. A, Near-field s-SNOM measurements of LiV_2O_5 PhPs (s_3) launched by a circular nanoantenna at $\omega = 997$ cm^{-1} (left), $\omega = 1001$ cm^{-1} (middle) and $\omega = 1006$ cm^{-1} (right). The diameter of the nanoantenna is ~ 2.5 μm, and its height is ~ 40 nm. The thickness of the flake is $d = 213$ nm. **B**, Same images as in **A** but with dashed gray lines added as guides to the eye.

Supplementary references

1. Álvarez-Pérez, G. *et. al.* Infrared Permittivity of the Biaxial van der Waals Semiconductor α-MoO_3 from Near- and Far-Field Correlative Studies. *Advanced Materials* **32**, 29 (2020).
2. Ma, W. *et al.* In-plane anisotropic and ultra-low-loss polaritons in a natural van der Waals crystal. *Nature* **562**, 557-562 (2018).
3. Aguilar-Merino, P. *et al.* Extracting the infrared permittivity of SiO_2 substrates locally by near-field imaging of phonon polaritons in a van der Waals crystal. *Nanomaterials* **11**, 120 (2021).
